# Supplementary material for: Is Formal Social Participation Associated with Cognitive Function in Middle-Aged and Older Adults? A Systematic Review with Meta-Analysis of Longitudinal Studies
Source: Behav Sci (Basel). 2024 Mar 22;14(4):262. doi: 10.3390/bs14040262 (PMC11047523; doi:10.3390/bs14040262)
Supplement: Supplementary file 1 [file behavsci-14-00262-s001.zip › behavsci-2855283-supplementary.pdf]

Is formal social participation associated with cognitive function in middle-aged and older adults? A systematic review with meta-analysis of longitudinal studies – Supplemental Material

Table S1. PRISMA checklist.

Section and Topic	Item #	Checklist item	Reported on page #
**TITLE**			
Title	1	Identify the report as a systematic review.	Page 1.
**ABSTRACT**			
Abstract	2	See the PRISMA 2020 for Abstracts checklist.	Page 1.
**INTRODUCTION**			
Rationale	3	Describe the rationale for the review in the context of existing knowledge.	Pages 2.
Objectives	4	Provide an explicit statement of the objective(s) or question(s) the review addresses.	Pages 2.
**METHODS**			
Eligibility criteria	5	Specify the inclusion and exclusion criteria for the review and how studies were grouped for the syntheses.	Pages 2 and 3.
Information sources	6	Specify all databases, registers, websites, organisations, reference lists and other sources searched or consulted to identify studies. Specify the date when each source was last searched or consulted.	Page 3.
Search strategy	7	Present the full search strategies for all databases, registers and websites, including any filters and limits used.	Supplemental material, Table S2, page 4.
Selection process	8	Specify the methods used to decide whether a study met the inclusion criteria of the review, including how many reviewers screened each record and each report retrieved, whether they worked independently, and if applicable, details of automation tools used in the process.	Pages 2 and 3.
Data collection process	9	Specify the methods used to collect data from reports, including how many reviewers collected data from each report, whether they worked independently, any processes for obtaining or confirming data from study investigators, and if applicable, details of automation tools used in the process.	Page 3.
Data items	10a	List and define all outcomes for which data were sought. Specify whether all results that were compatible with each outcome domain in each study were sought (e.g. for all measures, time points, analyses), and if not, the methods used to decide which results to collect.	Pages 2 and 3.
	10b	List and define all other variables for which data were sought (e.g. participant and intervention characteristics, funding sources). Describe any assumptions made about any missing or unclear information.	Pages 2 and 3.
Study risk of bias assessment	11	Specify the methods used to assess risk of bias in the included studies, including details of the tool(s) used, how many reviewers assessed each study and whether they worked independently, and if applicable, details of automation tools used in the process.	Pages 3.
Effect measures	12	Specify for each outcome the effect measure(s) (e.g. risk ratio, mean	Page 4.

Section and Topic	Item #	Checklist item	Reported on page #
		difference) used in the synthesis or presentation of results.	
Synthesis methods	13a	Describe the processes used to decide which studies were eligible for each synthesis (e.g. tabulating the study intervention characteristics and comparing against the planned groups for each synthesis (item #5)).	Pages 2, 3 and 6-9.
	13b	Describe any methods required to prepare the data for presentation or synthesis, such as handling of missing summary statistics, or data conversions.	Page 3 and 4.
	13c	Describe any methods used to tabulate or visually display results of individual studies and syntheses.	___
	13d	Describe any methods used to synthesise results and provide a rationale for the choice(s). If meta-analysis was performed, describe the model(s), method(s) to identify the presence and extent of statistical heterogeneity, and software package(s) used.	Pages 3 and 4.
	13e	Describe any methods used to explore possible causes of heterogeneity among study results (e.g. subgroup analysis, meta-regression).	Page 4.
	13f	Describe any sensitivity analyses conducted to assess robustness of the synthesised results.	Pages 4.
Reporting bias assessment	14	Describe any methods used to assess risk of bias due to missing results in a synthesis (arising from reporting biases).	Page 3 and Supplemental material, Table S3, page 5.
Certainty assessment	15	Describe any methods used to assess certainty (or confidence) in the body of evidence for an outcome.	Page 4.
**RESULTS**			
Study selection	16a	Describe the results of the search and selection process, from the number of records identified in the search to the number of studies included in the review, ideally using a flow diagram.	Pages 4 and 5.
	16b	Cite studies that might appear to meet the inclusion criteria, but which were excluded, and explain why they were excluded.	Supplemental material, Table S4, page 6-20.
Study characteristics	17	Cite each included study and present its characteristics.	Pages 6-9.
Risk of bias in studies	18	Present assessments of risk of bias for each included study.	Page 10.
Results of individual studies	19	For all outcomes, present, for each study: (a) summary statistics for each group (where appropriate) and (b) an effect estimate and its precision (e.g. confidence/credible interval), ideally using structured tables or plots.	Pages 11 and 12.
Results of syntheses	20a	For each synthesis, briefly summarise the characteristics and risk of bias among contributing studies.	Pages 5, 10 and 11.
	20b	Present results of all statistical syntheses conducted. If meta-analysis was done, present for each the summary estimate and its precision (e.g. confidence/credible interval) and measures of statistical heterogeneity. If comparing groups, describe the direction of the effect.	Pages 11 and 12.
	20c	Present results of all investigations of possible causes of heterogeneity among study results.	Pages 12.
	20d	Present results of all sensitivity analyses conducted to assess the robustness of the synthesised results.	Pages 11, 12 and

Section and Topic	Item #	Checklist item	Reported on page #
			supplemental material, Table S5, page 21.
Reporting biases	21	Present assessments of risk of bias due to missing results (arising from reporting biases) for each synthesis assessed.	Page 10.
Certainty of evidence	22	Present assessments of certainty (or confidence) in the body of evidence for each outcome assessed.	Pages 11 and 12.
**DISCUSSION**			
Discussion	23a	Provide a general interpretation of the results in the context of other evidence.	Pages 13 and 14.
	23b	Discuss any limitations of the evidence included in the review.	Page 14.
	23c	Discuss any limitations of the review processes used.	Pages 14.
	23d	Discuss implications of the results for practice, policy, and future research.	Page 14.
**OTHER INFORMATION**			
Registration and protocol	24a	Provide registration information for the review, including register name and registration number, or state that the review was not registered.	Page 2.
	24b	Indicate where the review protocol can be accessed, or state that a protocol was not prepared.	Page 2.
	24c	Describe and explain any amendments to information provided at registration or in the protocol.	___
Support	25	Describe sources of financial or non-financial support for the review, and the role of the funders or sponsors in the review.	Page 15.
Competing interests	26	Declare any competing interests of review authors.	Page 15.
Availability of data, code and other materials	27	Report which of the following are publicly available and where they can be found: template data collection forms; data extracted from included studies; data used for all analyses; analytic code; any other materials used in the review.	Page 15.

Table S2. Database search strategy for each database included.

Database	Search strategy
PubMed	(((((((((((((((((((((((((formal social[Title/Abstract]) OR (social participation[Title/Abstract])) OR (social engage*[Title/Abstract])) OR (activity participation[Title/Abstract])) OR (social activ*[Title/Abstract])) OR (group[Title/Abstract])) OR (social capital[Title/Abstract])) OR (leisure[Title/Abstract])) OR (social disengage*[Title/Abstract])) OR (socially productive active*[Title/Abstract])) OR (organiz*[Title/Abstract])) OR (club*[Title/Abstract])) OR (association*[Title/Abstract])) OR (volunteer*[Title/Abstract])) OR (gatherings[Title/Abstract])) AND (cognitive decline[Title/Abstract])) OR (cognitive ageing[Title/Abstract])) OR (cognit* loss[Title/Abstract])) OR (cognit* change[Title/Abstract])) OR (cognitive function[Title/Abstract])) OR (cognitive performance[Title/Abstract])) OR (cognition[Title/Abstract])) AND (older*[Title/Abstract])) OR (elder*[Title/Abstract])) OR (middle age[Title/Abstract])) AND (longitudinal[Title/Abstract]).
Scopus	(((((((((((((((((((((((((TS=(formal social)) OR TS=(social participation)) OR TS=(social engage*)) OR TS=(activity participation)) OR TS=(social activ*)) OR TS=(group)) OR TS=(social capital)) OR TS=(leisure)) OR TS=(social disengage*)) OR TS=(socially productive active*)) OR TS=(organiz*)) OR TS=(club*)) OR TS=(association*)) OR TS=(volunteer*)) OR TS=(gatherings)) AND TS=(cognitive decline)) OR TS=(cognitive ageing)) OR TS=(cognit* loss)) OR TS=(cognit* change)) OR TS=(cognitive function)) OR TS=(cognitive performance)) OR TS=(cognition)) AND TS=(older*)) OR TS=(elder*)) OR TS=(middle age)) AND TS=(longitudinal).
Web of Science	TITLE-ABS-KEY ("formal social") OR TITLE-ABS-KEY ("social participation") OR TITLE-ABS-KEY ("social engage*") OR TITLE-ABS-KEY ("activity participation") OR TITLE-ABS-KEY ("social activ*") OR TITLE-ABS-KEY ("group") OR TITLE-ABS-KEY ("social capital") OR TITLE-ABS-KEY ("leisure") OR TITLE-ABS-KEY ("social disengage*") OR TITLE-ABS-KEY ("socially productive active*") OR TITLE-ABS-KEY ("organiz*") OR TITLE-ABS-KEY ("club*") OR TITLE-ABS-KEY ("association*") OR TITLE-ABS-KEY ("volunteer*") OR TITLE-ABS-KEY ("gatherings") AND TITLE-ABS-KEY ("cognitive decline") OR TITLE-ABS-KEY ("cognitive ageing") OR TITLE-ABS-KEY ("cognit* loss") OR TITLE-ABS-KEY ("cognit* change") OR TITLE-ABS-KEY ("cognitive function") OR TITLE-ABS-KEY ("cognitive performance") OR TITLE-ABS-KEY ("cognition") AND TITLE-ABS-KEY ("older*") OR TITLE-ABS-KEY ("elder*") OR TITLE-ABS-KEY ("middle age") AND TITLE-ABS-KEY ("longitudinal") AND PUBYEAR > 2009 AND PUBYEAR < 2023.

Table S3. Appraisal of risk of bias criteria defined according to Risk of Bias Assessment tool for Non-Randomized Studies (RoBANS) tool.

Domain	Description
**Selection of participants**	**Selection bias** caused by inadequate selection of participants.<br><br>The selection of participants should be clearly specified and defined.<br>As a high risk, we considered:<br>(i) participants selected or recruited from different populations, and/or (ii) participants selected from different time periods, and/or (iii) using a restricted group of individuals without a reasonable and valid explanation.
**Confounding variables**	**Selection bias** caused by inadequate confirmation and consideration of confounding variables.<br><br>The sampling design should be appropriate and representative of the target population. The criteria used to select participants for the studies should be relevant and appropriate for the research question.<br>Potential confounding variables should be measured and adjusted statistically for their impact on the relationship between exposure (formal social participation) and outcome (cognitive function). We considered that studies should at least control for the following variables: (i) age, (ii) sex, (iii) education, (iv) income, (v) employment status, (vi) physical health, and (vii) mental health.
**Exposure measurement**	**Performance bias** caused by inadequate measurement of exposure.<br><br>We classified studies as having a high risk of bias if they exhibited the following characteristics: (i) poorly defined, invalid, or unreliable measures of formal social participation, and/or (ii) inconsistent implementation of the formal social participation measure across study participants, with variations in content or timing between respondents, and/or (iii) failure to examine different levels of formal social participation in relation to cognitive function, and/or (iv) insufficient timeframe, where it was unlikely to observe an association between formal social participation and cognitive function even if it existed, and/or (v) lack of repeated assessment of formal social participation over time.
**Blinding outcome assessment**	**Detection bias** caused by inadequate blinding of outcome assessment.<br><br>The outcome assessors should be blinded to the exposure status of participants.<br>Classified as unclear when self-reported measures of the outcome were employed. As per Cochrane recommendations, assessments relying on self-reported measures should always be evaluated with some concerns regarding blinding. Since the RoBANS tool does not have a specific category to address this concern, we classified it as unclear.
**Incomplete outcome data**	**Attrition bias** caused by inadequate handing of incomplete data outcome.<br><br>We classified studies as having a high risk of bias if the loss to follow-up at baseline was more than 20%.
**Selective outcome reporting**	**Reporting bias** caused by selective outcome reporting.<br><br>Studies were classified as having a high risk of bias if they met one or more of the following criteria: (i) the authors selectively analyzed only a subset of outcomes or a subset of the study sample without providing proper justification, (ii) reasons for missing data were not reported, (iii) qualitative reporting lacked supporting evidence from the data, (iv) the methods section did not provide information on how the analyses would be conducted, (v) the methods section did not provide information on how the outcomes would be assessed, (vi) there was a contradiction between the methods described and the reported results.

Table S4. Sensitivity analysis for the association between formal social participation and cognitive function.

Outcome	Main findings			Sensitivity analyses			
	k, n	I^2, p-value	OR (95% CI) [95% PI]	k, n	I^2, p-value	OR (95% CI) [95% PI]	Excluded study [reasons]
Cognitive function (dichotomous)	6, 33489	0%, p=0.45	**0.78 (0.75 to 0.82) [0.73 to 0.84]**	5, 20227	0%, p=0.51	**0.82 (0.75 to 0.89) [0.73 to 0.92]**	Infurna et al. (2016) [43] [large sample size; single activity focused].
				5, 25921	4%, p=0.39	**0.78 (0.74 to 0.81) [0.73 to 0.82]**	Kim (2020) [25] [large sample size; lowest mean age].
				4, 12659	6%, p=0.37	**0.82 (0.73 to 0.93) [0.65 to 1.04]**	Infurna et al. (2016) [43] and Kim (2020) [25] [reasons above].
				4, 28549	19%, p=0.29	**0.81 (0.75 to 0.87) [0.66 to 0.99]**	Min (2018) [24] and Kim et al. (2017) [44] [partial overlapping of sample].

Notes: k=number of studies; n=number of participants; I^2=Heterogeneity; OR=Odds Ratio; CI=Confidence Interval; PI=Prediction Interval.
